# Recent Advances in Magnetic Two-Dimensional van der Waals Heterostructures: Synthesis, Properties, and Spintronic Applications: A Review

**DOI:** 10.3390/nano15201569

**Published:** 2025-10-15

**Authors:** Meri Algarni

**Affiliations:** Department of Physics, Faculty of Science, Al-Baha University, Alaqiq 65779-7738, Saudi Arabia; maalgarni@bu.edu.sa

**Keywords:** two-dimensional van der Waals materials, ferromagnetism, van der Waals heterostructures, magnetic properties, spintronic device applications

## Abstract

Two-dimensional (2D) van der Waals (vdW) magnetic materials have emerged as a frontier in condensed matter physics and materials science, offering unprecedented opportunities for next-generation spintronic technologies. This review examines the synthesis, properties, and transport phenomena of 2D magnetic materials, with particular emphasis on their integration into spintronic devices. A comprehensive historical overview of magnetic materials is provided, tracing the evolution of intrinsic ferromagnetism in the 2D limit, highlighting key materials such as Cr_2_Ge_2_Te_6_, Fe_3_GeTe_2_, and CrI_3_. Special attention is devoted to the fundamental magnetic properties—including magnetic anisotropy, Curie temperature, and spin polarization—that underpin their functional performance. Major synthesis strategies are evaluated, including chemical vapor deposition, micromechanical exfoliation, and molecular beam epitaxy, focusing on scalability, interface control, and material purity. Furthermore, hallmark transport phenomena are discussed, such as giant magnetoresistance, the quantum anomalous Hall effect, spin–orbit torque, and the role of exchange bias and skyrmions in vdW heterostructures. Throughout the review, current limitations, unresolved questions, and emerging research directions are identified that will accelerate the deployment of 2D magnetic materials in flexible, reconfigurable, and quantum spintronic systems. This work aims to guide ongoing experimental and theoretical efforts and articulate a vision for advancing the field toward device-level implementation.

## 1. Introduction

The advent of two-dimensional (2D) materials has transformed materials science and nanotechnology, beginning with the groundbreaking isolation of graphene—a monolayer of carbon atoms arranged into a hexagonal lattice [1,2]. This milestone triggered a global surge in the discovery and synthesis of other 2D compounds with diverse physical properties, including transition metal dichalcogenides, hexagonal boron nitride (h-BN), and layered metal oxides [3]. Among these developments, the identification of intrinsic ferromagnetism in several 2D materials has attracted significant interest, particularly for applications in spintronics—a field that exploits the electron’s spin degree of freedom in addition to its charge [4].

The scientific understanding of magnetism has evolved substantially since antiquity, when natural magnetism was first observed in lodestone, an iron oxide mineral. The discovery of ferromagnetism in iron and its alloys in the 19th century marked a critical advancement [5,6,7], while the advent of quantum mechanics in the 20th century provided a robust theoretical foundation that enabled the development of modern magnetic materials underpinning today’s spintronic and electronic technologies [8].

The discovery of intrinsic magnetism in 2D materials represents a paradigm shift for spintronics. Ferromagnetic materials such as Cr_2_Ge_2_Te_6_ (CGT), Fe_3_GaTe_2_ (F_3_GT), CrTe_2_, Fe_5_GeTe_2_ (F_5_GT), CrI_3_ and graphene-based heterostructures exhibit magnetic properties that are highly tunable at the atomic scale, making them attractive candidates for next-generation nanoelectronic applications [9,10]. These materials offer transformative potential for practical applications including non-volatile magnetic memory, reconfigurable logic circuits, ultra-sensitive magnetic sensors, and quantum information processing platforms. A landmark achievement occurred in 2017, when researchers experimentally demonstrated 2D ferromagnetism in a monolayer of CrI_3_, confirming that magnetic order can be sustained even in atomically thin layers and establishing CrI_3_ as a prototype system for investigating spintronic phenomena, including giant tunneling magnetoresistance and voltage-controlled magnetic switching [11].

Within the broader spintronic landscape, 2D van der Waals (vdW) magnetic materials offer distinct advantages over conventional platforms. Unlike heavy metals, magnetic oxides, Heusler alloys, and topological insulators, vdW heterostructures provide atomically sharp interfaces that eliminate lattice-matching constraints, layer-by-layer control over magnetic coupling, and unprecedented electrical tunability through gate voltages—capabilities that enable direct manipulation of magnetic ordering at the atomic scale. This review focuses specifically on recent advances in magnetic 2D vdW heterostructures; comprehensive cross-platform comparisons are available in recent reviews [12,13,14].

The magnetic behavior of 2D systems represents an intermediate regime between three-dimensional (3D) and one-dimensional (1D) systems. While 3D materials support magnetic phase transitions at finite temperatures, long-range magnetic order in 1D systems is theoretically restricted to absolute zero (T = 0). In contrast, 2D systems can support finite-temperature magnetic ordering under specific conditions—particularly when anisotropy and other stabilizing factors are present [6]. These materials not only offer technological relevance but also provide a platform for exploring exotic magnetic phenomena in reduced dimensions, including exchange bias effects through interfacial coupling with antiferromagnetic materials and voltage-controlled magnetic phase transitions [15].

A longstanding theoretical constraint, the Mermin–Wagner theorem, suggested that long-range magnetic order could not exist in 2D systems at finite temperatures due to thermal fluctuations. However, the discovery of materials like CGT and F_3_GT, which exhibit robust ferromagnetic ordering down to the monolayer limit, has challenged this view [9]. These findings have opened new opportunities for fabricating ultracompact spintronic devices with properties and functionalities unattainable in traditional 3D systems. However, significant challenges remain, including achieving consistent room temperature operation, ensuring long-term stability under ambient conditions, and developing scalable synthesis techniques compatible with industrial manufacturing.

The central question addressed in this review is how the intrinsic physical characteristics of 2D ferromagnetic materials, including magnetic anisotropy, Curie temperatures (T_C_), spin polarization, and exchange coupling—can be harnessed for practical spintronic applications, including magnetic memory, logic devices, and quantum technologies. This review examines fundamental properties governing 2D magnetism in ferromagnetic systems, surveys key synthetic approaches enabling material fabrication, analyzes electronic and transport phenomena, and identifies pathways toward device implementation. Particular emphasis is placed on Fe-Ge-Te systems due to their near-room temperature operation and demonstrated device performance, while also examining other promising materials including CrI_3_ for tunnel junction applications and CrTe_2_ for ambient-condition spintronics.

This review is organized as follows: Section 2 examines the intrinsic properties of key 2D ferromagnetic materials, Section 3 discusses synthesis techniques for producing high-quality materials, Section 4 analyzes electronic and magnetic structures, Section 5 explores transport phenomena and device applications, and Section 6 provides concluding remarks and future perspectives for the field.

## 2. Intrinsic Properties of 2D Magnetic Materials

2D magnetic materials exhibit intrinsic magnetic ordering in structures composed of one to a few atomic layers, enabling novel magnetic phenomena distinct from their 3D counterparts [6]. Their reduced dimensionality allows them to retain magnetism at the nanoscale, making them attractive for applications in next-generation spintronic and quantum devices [16,17]. The magnetic behavior of these materials is governed by their atomic configurations, particularly the arrangement of transition metal ions in layered lattices, which induce magnetic anisotropy and spin polarization. Representative examples include CGT with a T_C_ of approximately 61 K and F_3_GT with T_C_ values reaching 310 K—approaching room temperature operation [2,18].

The field of 2D magnetism gained momentum following the isolation of graphene in 2004 and witnessed a major breakthrough in 2017 with the observation of intrinsic ferromagnetism in monolayer CrI_3_ and bilayer CGT [9,10,19]. These materials typically possess strong intralayer exchange interactions, coupled with spin–orbit coupling (SOC) and crystal field effects, which collectively determine their T_C_ and magnetic stability. Such intrinsic features are essential for developing spintronic devices operating under technologically viable conditions.

Structurally, 2D magnetic materials are composed of layers strongly bonded through covalent or ionic interactions, while adjacent layers are held together by weak vdW forces [1,20]. This characteristic enables mechanical exfoliation into monolayer or few-layer systems, facilitating device integration [21]. For example, both intra- and interlayer magnetic interactions influence spin alignment and overall magnetic behavior, making the control of interfacial coupling a critical design factor [6,7,15].

Magnetic anisotropy, arising primarily from SOC, plays a vital role in stabilizing long-range magnetic order in low-dimensional systems. In materials like CrI_3_, strong perpendicular anisotropy ensures out-of-plane magnetization, enabling robust ferromagnetism even in monolayers [22,23]. However, due to the intrinsic limitations of reduced dimensionality, 2D magnets generally exhibit lower T_C_ values compared to their bulk analogues. For instance, monolayer CrI_3_ displays a T_C_ of ~45 K, while multilayer F_3_GT exhibits values around 220–310 K depending on thickness and external modulation [22,24]. Consequently, efforts to enhance T_C_ through strain engineering, doping, and heterostructure design remain an active area of research for realizing room temperature spintronic devices.

Among the key intrinsic properties that make 2D magnetic materials highly desirable for spintronics is spin polarization, which refers to the preferential alignment of electron spins along a specific direction [25]. This property is essential for manipulating spin currents in spintronic circuits. In materials like CrI_3_, strong intralayer exchange interactions yield highly spin-polarized electronic bands, which enhance device performance [22,23]. These interactions also influence the electronic band structure, where SOC—the coupling between an electron’s spin and its orbital motion—introduces further splitting and anisotropy. SOC is crucial for phenomena such as the quantum anomalous Hall effect (QAHE), where quantized Hall conductance arises without external magnetic fields.

Another fundamental feature of 2D magnets is the presence of spin waves, or magnons, which are quantized collective excitations of electron spins [15]. These magnons enable the transmission of information without electrical current, making them highly relevant for magnonic devices aimed at low-power spin-based communication. Additionally, 2D magnets can exhibit magnetic domain structures, where regions of uniform magnetization are separated by domain walls. The size and dynamics of these domains are governed by the balance between magnetic anisotropy and exchange interactions [7,26]. In systems like CrI_3_, external electric or magnetic fields can manipulate domain patterns, offering promising prospects for domain wall-based memory and logic devices.

Furthermore, defects and edge states can profoundly influence the magnetic properties of 2D materials. Point defects, vacancies, and dopants can locally modify magnetic moments, while edge states in topologically non-trivial systems may host spin-polarized channels suitable for spin filtering [27]. These features expand the design space for customizing 2D magnetic behaviors for targeted applications.

The magnetic behavior of 2D systems can also be externally tuned via strain, electric fields, or magnetic stimuli. For instance, strain engineering can modulate lattice symmetry and bonding angles, thereby influencing magnetic anisotropy and T_C_. In F_3_GT, applied strain has been shown to substantially increase T_C_ and magnetization [24,28]. Similarly, gate-controlled electric fields can dynamically modulate interlayer coupling and magnetic ordering [29,30]. Such tunability is critical for realizing adaptive and energy-efficient spintronic components.

Several 2D ferromagnetic materials have been experimentally realized, each exhibiting distinctive structural and magnetic characteristics that make them suitable for integration into spintronic devices. Below is a concise overview of the most studied materials:

### 2.1. Cr_2_Ge_2_Te_6_ (CGT)

The Cr_2_Ge_2_Te_6_ crystal was initially produced through a solid-state synthesis approach [31]. In its bulk form, CGT exhibits semiconducting ferromagnetic behavior, characterized by a bandgap of approximately 0.7 eV and a T_C_ around 61 K. As a representative 2D magnetic semiconductor, CGT exhibits robust ferromagnetic coupling both within and between its vdW layers. Beyond its intrinsic magnetic ordering, CGT has attracted attention for its tunable magnetic properties in low-dimensional devices.

Zhang and colleagues demonstrated that magnetism in few-layer CGT can be modulated by an external electric field, revealing its potential for gate-tunable spintronic applications [32]. Moreover, the magnetic behavior of CGT has been probed electrically using the anomalous Hall effect (AHE) in heterostructures. Lohmann et al. (2019) induced an AHE signal in adjacent platinum layers to detect magnetism in insulating CGT, while Xiang et al. (2017) explored electric field effects in multilayer CGT and reported significant resistance changes at approximately ±0.2 T in gated devices near the ferromagnetic–metallic transition region [9,33]. However, direct electrical transport measurements in ungated CGT remain challenging due to its high intrinsic resistance below the T_C_.

**Figure 1:**
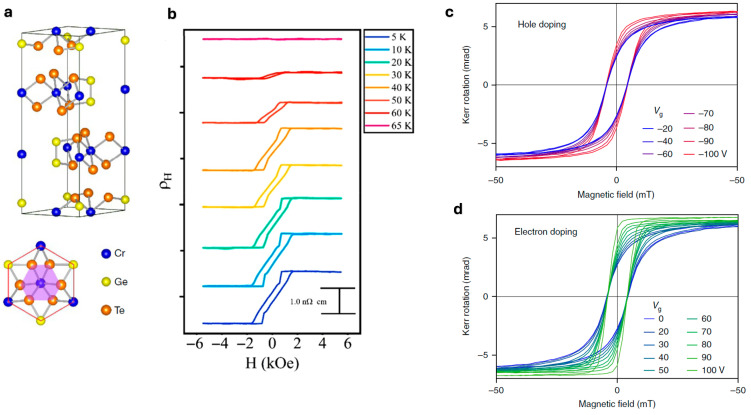
Characterization of CGT crystals. (**a**) Crystal structure (side and top views) of CGT. Bulk CGT has a layered structure with interlayer vdW spacing of 3.4 Å. Reprinted with permission from ref. [9]. (**b**) AHE hysteresis loops for select temperatures from 65 to 5 K after subtraction of the linear ordinary Hall background. Reprinted with permission from ref. [33]. (**c**,**d**) Kerr rotation angles for CGT under hole doping (**c**) and electron doping (**d**) at 40 K. Reprinted with permission from ref. [32].

As shown in Figure 1a, bulk CGT exhibits a layered crystal structure with an interlayer vdW spacing of approximately 3.4 Å [9]. Leveraging its vdW nature, Lohmann et al. (2019) fabricated CGT/Pt heterostructure devices with CGT thicknesses reduced to ~35 nm, enabling the detection of magneto-transport signatures induced in the Pt layer. Figure 1b presents AHE measurements in Pt, recorded between 5 and 65 K with a 2.0 mA excitation current under out-of-plane magnetic fields, where the hysteresis loops were obtained after subtracting the linear ordinary Hall background [9].

Wang et al. (2018) demonstrated that few-layer ferromagnetic semiconducting CGT can operate as transistors, with electrostatic gating effectively modulating their magnetic state. This gate-induced tuning, observed through micro-area Kerr effect (MOKE) measurements performed below T_C_, underscores the potential of CGT for electrically adjustable spintronic devices. As shown in Figure 1c,d, the MOKE hysteresis loops were clearly modified below T_C_ through both electron and hole doping [32].

### 2.2. Fe_3_GaTe_2_ (F_3_GT)

Fe_3_GaTe_2_, an iron-based telluride, has emerged as a robust 2D ferromagnet with a relatively high T_C_ (~220 K in bulk), making it suitable for near room temperature operations. It exhibits strong ferromagnetic exchange coupling and is amenable to exfoliation into thin layers. Criticality analysis estimates the magnetic coupling length to span approximately five vdW layers, supporting its integration into magnetic vdW heterostructures [34]. Structurally, F_3_GT is a quasi-2D itinerant ferromagnet composed of Fe_3_Ge heterometallic slabs sandwiched between two Te layers, forming a characteristic vdW layered structure.

Various fabrication approaches have been developed to isolate few-layer or monolayer F_3_GT for device integration. Mechanical exfoliation has proven effective in producing atomically thin F_3_GT on SiO_2_/Si substrates [24], while an alternative Al_2_O_3_-assisted exfoliation method was introduced by Deng et al. to reliably obtain monolayer F_3_GT flakes [29]. These techniques have enabled detailed exploration of the thickness-dependent magnetic behavior of F_3_GT nanoflakes.

Tan et al. (2018) examined the AHE in single-crystalline F_3_GT and observed that its magnetic response varied significantly with thickness [35]. When the thickness is reduced below 200 nm, the nanoflakes exhibit a hard magnetic phase with pronounced coercivity and a nearly square-shaped hysteresis loop. These properties make F_3_GT highly suitable for integration into vdW-based spintronic devices, including magnetic memory and logic components.

**Figure 2:**
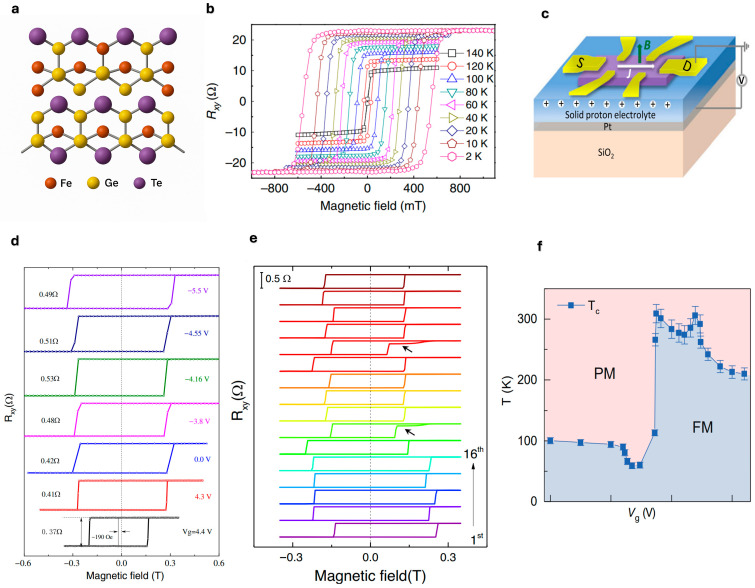
A comprehensive overview of the structural and magnetic properties of F_3_GT nanosheets. (**a**) The side view of the bilayer F_3_GT crystal structure illustrates the atomic arrangement of Fe (red), Ge (blue), and Te (yellow) atoms within the vdW lattice. (**b**) $R_{xy}$*(B)* measurements performed on a 10.4 nm-thick F_3_GT flake reveal distinct hysteresis loops across a temperature range from 2 K to 140 K, indicating robust and stable ferromagnetic ordering [35]. (**c**,**d**) A schematic depiction of a Hall-bar device fabricated on a rigid proton substrate using a 115 nm-thick F_3_GT flake is presented. The gate voltage varied between 5.5 V and +4.4 V, enabling tunable modulation of the ferromagnetic response. This gating systematically reduced both magnetization and coercivity. Reprinted with permission from ref. [30]. (**e**) The EB effect observed in bilayer F_3_GT following zero-field cooling to 2 K exhibits stochastic variations in coercivity and bias fields across successive hysteresis measurements. Reprinted with permission from ref. [30]. (**f**) A temperature-and gate-voltage-dependent phase diagram showing paramagnetic to ferromagnetic transition with T_C_ elevation toward room temperature through ionic gating. Reprinted with permission from ref. [29].

As illustrated in Figure 2a, a side view of the bilayer F_3_GT crystal structure reveals the atomic arrangement of iron (Fe), germanium (Ge), and tellurium (Te) atoms within the vdW lattice. The presence of intrinsic ferromagnetism in exfoliated F_3_GT flakes is confirmed in Figure 2b through AHE resistance $R_{xy}$*(B)* measurements, which display well-defined hysteresis loops over a wide temperature range (2 K to 140 K), indicating robust magnetic order even at reduced thickness [35].

Zheng et al. (2020) reported that electrostatic gating can effectively tune the magnetic behavior of F_3_GT. This was achieved by fabricating Hall-bar devices on solid protonic substrates and varying the gate voltage between −5.5 V and +4.4 V (Figure 2c,d) [30]. The findings showed that increased proton intercalation within the vdW layers strengthens the interlayer magnetic coupling. Furthermore, the study identified the occurrence of exchange bias (EB) phenomena in bilayer F_3_GT after zero-field cooling, accompanied by random changes in both coercivity and loop displacement over successive magnetic hysteresis loops (Figure 2e) [30].

Similarly, Deng et al. (2018) demonstrated that applying an ionic gate to ultrathin F_3_GT flakes can raise the T_C_ to nearly room temperature (~300 K), as illustrated in Figure 2f. This modulation arises from gate-induced electron doping, which alters the electronic band structure and density of states at the Fermi level. According to the Stoner model for itinerant ferromagnetism, these changes in the electronic structure directly influence the magnetic exchange interactions, enabling the enhancement of ferromagnetic ordering and elevation of the T_C_. The phase diagram shows the transition from paramagnetic to ferromagnetic phases as a function of both temperature and gate voltage, demonstrating the potential for voltage-controlled magnetoelectronic devices [29].

### 2.3. Fe_5_GeTe_2_ (F_5_GT)

Fe_5_GeTe_2_ is emerging as a compelling candidate for next-generation spintronic technologies due to its combination of structural versatility and robust magnetic behavior. Crystallographically, Fe_5−X_GT adopts a hexagonal tourmaline-type structure, which forms weakly bonded vdW layers—facilitating mechanical exfoliation and enabling integration into 2D device architectures [36]. One of its key features is a high T_C_, ranging between 270 K and 310 K in bulk single crystals. Ferromagnetic ordering persists in mechanically exfoliated flakes as thin as ~12 nm (approximately four unit cells), though T_C_ in such exfoliated samples typically ranges from 270 to 300 K, with no systematic thickness dependence in the 10–30 nm range. The observed T_C_ variation reflects sensitivity to processing conditions, thermal cycling, and intrinsic sample-to-sample differences [37].

Beyond its intrinsic magnetism, F_5_GT displays a complex and tunable magnetic phase diagram. When incorporated into vdW heterostructures such as FePS_3_/F_5_GT, it exhibits a pronounced exchange bias effect that can be modulated by adjusting the thickness of the ferromagnetic layers [38,39]. This thickness-dependent interfacial coupling emphasizes the material’s promise for reconfigurable spintronic architectures. Additionally, Tan et al. (2021) demonstrated that F_5_GT undergoes a gate-controlled magnetic phase transition, where AHE measurements under varying thicknesses and gate voltages revealed that the magnetic ground state is highly sensitive to the doping level [40].

**Figure 3:**
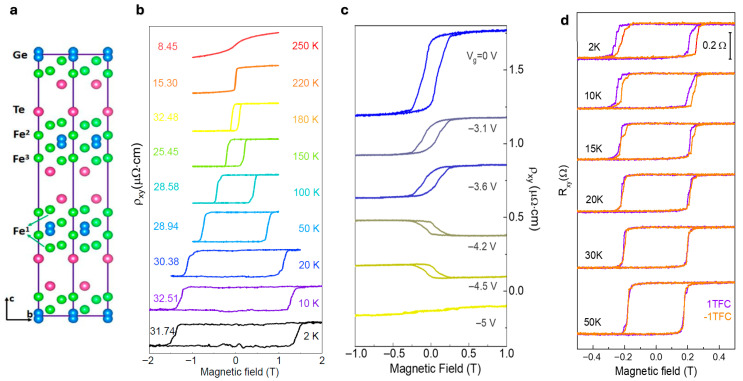
Crystal structure and magnetic properties of F_5_GT thin film. (**a**) Illustration of the F_5_GT crystal lattice as viewed along the *a*-axis, highlighting the layered vdW stacking of Fe, Ge, and Te atoms. Reprinted with permission from ref. [40]. (**b**) Magnetic-field-dependent $\rho_{xy}$(B) measurements for a 6.8 nm-thick F_5_GT device recorded over a temperature range from 2 K to 250 K, demonstrating the evolution of magnetic hysteresis with thermal variation. Reprinted with permission from ref. [40]. (**c**) $\rho_{xy}$(B) hysteresis loops obtained at multiple gate voltages for a 40 nm-thick F_5_GT nanoflake, revealing gate-induced modulation of the Hall response. Reprinted with permission from ref. [40]. (**d**) EB phenomena observed in FePS_3_–F_5_GT vdW heterostructures across different temperatures from 2 K to 150 K in the absence of an applied gate voltage. Reprinted with permission from ref. [39].

As illustrated in Figure 3a, the crystal lattice of F_5_GT viewed along the a-axis reveals the layered vdW stacking of Fe, Ge, and Te atoms [40]. The magnetic properties of F_5_GT are investigated in Figure 3b through magnetic-field-dependent AHE resistivity ($\rho_{xy}$) measurements performed on a 6.8 nm thick F_5_GT device over a temperature range of 2 K to 250 K. The numerical values displayed in the figure (8.45, 15.30, etc.) represent the $\rho_{xy}$ values in μΩ·cm at each measurement temperature, indicating the strength of the ferromagnetic ordering under different thermal conditions. These values demonstrate that the AHE is significantly enhanced in ultrathin F_5_GT compared to thicker samples, reflecting the enhanced magnetic properties in the nanoscale regime. The results reveal a systematic evolution of hysteresis loops with increasing temperature, underscoring the pronounced thermal modulation of the ferromagnetic order [40].

Gate-induced magnetic modulation is illustrated in Figure 3c, where the $\rho_{xy}$*(B)* hysteresis loops of a 40 nm-thick F_5_GT nanoflake progressively shrink with increasingly negative gate voltages, leading to near-complete suppression of ferromagnetic order at V_g_ = −5 V. Notably, the anomalous Hall resistivity not only decreases but also reverses sign at −4.2 V and −4.5 V, with the loop vanishing entirely at −5 V, suggesting a possible magnetic phase transition [40].

Building on the concept of electrically tuning magnetic order, Figure 3d highlights electrically controllable EB in a FePS_3_– F_5_GT vdW heterostructure, where the EB effect can be reversibly tuned via a solid protonic gate. The field-cooling measurements were performed under ±1T magnetic fields (denoted as “1TFC” for +1T field-cooling and “−1TFC” for −1T field-cooling) by cooling the sample from 150 K (above the Néel temperature of FePS_3_) down to the measurement temperature to establish the EB effect. This electrically controllable EB represents a significant step toward enabling vdW heterostructure-based magnetic logic for future low-energy electronics [39]. Extending this protonic gating approach to even thinner systems, Tan et al. (2021) demonstrated that applying similar gating techniques to ~5.4 nm-thick F_5_GT nanosheets enables ultrahigh electron doping (~10^21^ cm^−3^), which drives a distinct magnetic phase transition from FM to AFM, further showcasing the versatility of electric-field control in 2D magnetic materials [40].

### 2.4. CrTe_2_

CrTe_2_ is a vdW layered transition-metal dichalcogenide that crystallizes in a trigonal lattice where hexagonally arranged chromium planes are confined between adjacent tellurium sheets. This structural motif not only underpins its stability as an atomically thin material but also gives rise to its intrinsic 2D ferromagnetism [41,42]. Bulk 1T–CrTe_2_ has been identified as a metallic ferromagnet with a T_C_ of approximately 310 K, indicating the stability of its magnetic ordering near room temperature [41].

A significant breakthrough was reported by Zhang et al. (2021), who successfully synthesized high-quality CrTe_2_ thin films via molecular beam epitaxy and confirmed the presence of intrinsic ferromagnetism at room temperature [43]. Sun et al. reported that ultra-thin vdW crystals of 1T-CrTe_2_ exhibit robust ferromagnetism persisting up to room temperature [44]. Building upon these findings, recent studies have demonstrated that mechanically exfoliated CrTe_2_ flakes with thicknesses down to ~10 nm also retain robust ferromagnetic behavior above room temperature. These thin flakes exhibit well-defined in-plane magnetic domains and Néel-type domain walls, supporting the material’s potential for ambient-condition spintronic applications [44,45].

**Figure 4:**
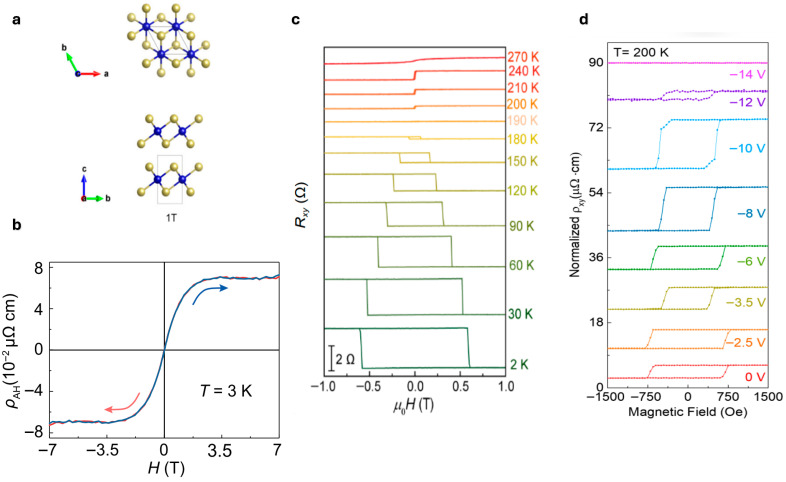
Electronic transport and -structural characterizations of 1T-CrTe_2_. (**a**) Top and side views of the atomic structure of bulk 1T -CrTe_2_, with chromium atoms represented by blue spheres and tellurium atoms by golden spheres. Reprinted with permission from ref. [46]. (**b**) $\rho_{xy}$ in a 170-nm 1T-CrTe_2_ nanoflake, showing clear out-of-plane magnetic field dependence at 3 K. Reprinted with permission from ref. [49]. (**c**) Temperature-dependent Hall *R_xy_* measurements for atomically thin (~16.0 nm) Cr_1−x_Te nanosheets under out-of-plane magnetic fields, demonstrating robust ferromagnetic transport in the ultrathin limit. Reprinted with permission from ref. [50]. (**d**) $\rho_{AHE}$ hysteresis loops under various gate voltages in a quasi-2D magnet Cr_1.2_Te_2_, showing gate-tunable magnetic transport. Reprinted with permission from ref. [51].

In addition to its magnetic robustness, CrTe_2_ possesses strong spin polarization and a thickness-dependent electronic band structure, positioning it as a promising candidate for 2D spintronic and magneto-optoelectronic devices [46,47]. As illustrated in Figure 4a, the atomic structure of bulk 1T-CrTe_2_ shows the characteristic layered arrangement [46]. Lingjia et al. confirmed that a robust AHE can be realized in 1T-CrTe_2_ vdW ferromagnetic single crystals synthesized using CVD [48].

With respect to magnetic transport, Meng et al. reported record anomalous Hall resistivity ($\rho_{AH}$) in a 170-nm vdW ferromagnetic 1T-CrTe_2_ nanoflake [49]. As shown in Figure 4b, the $\rho_{AH}$ of this flake exhibits clear dependence on the out-of-plane magnetic field at 3 K. Figure 4c displays electrical transport studies of Cr_1−x_Te nanosheets, revealing temperature-dependent Hall resistance ($R_{xy})$ measurements on a ~16.0 nm thick sample from 2 K to 270 K. At low temperatures (2–190 K), square-shaped $R_{xy}-\mu_{0}H$ hysteresis loops characteristic of the AHE are observed, indicating robust ferromagnetic ordering with strong perpendicular magnetic anisotropy. As temperature increases, the hysteresis loops progressively narrow and eventually vanish at approximately 190 K. Upon further warming above this critical temperature, new hysteresis loops emerge with reversed $R_{xy}$ polarity, signaling a magnetic phase transition from hard to soft ferromagnetic behavior [50].

Electrostatic control through protonic gating offers a versatile approach for tuning magnetic transport properties in Cr_1.2_Te_2_ nanoflakes, representing an important advancement in voltage-controlled spintronic devices. Tan et al. (2021) demonstrated electrical modulation of ferromagnetism in Cr_1.2_Te_2_ nanoflakes at temperatures extending to room temperature through protonic gate techniques [51]. As illustrated in Figure 4d, the evolution of anomalous Hall resistivity ($\rho_{AHE}$) hysteresis loops in a 43-nm-thick Cr_1.2_Te_2_ nanoflake shows systematic dependence on Vg. When Vg was swept from 0 to −10 V, the $\rho_{AHE}$ of the near-square-shaped loops increased by approximately threefold. Further negative gating beyond −10 V caused a sharp decline in the anomalous Hall response, with complete suppression of hysteresis behavior at Vg = −14 V. This gate-induced modulation corresponds to significant changes in carrier density, with hole concentration decreasing by 3.4 × 10^21^ cm^−3^ as proton intercalation increases throughout the voltage sweep from 0 to −14 V. The voltage-controlled modulation of magnetic properties and phase behavior in room temperature Cr_1.2_Te_2_ establishes a pathway toward next-generation spintronic technologies.

### 2.5. Graphene

Graphene, a single atomic layer of carbon atoms arranged in a hexagonal lattice, is widely regarded as the prototypical 2D material. While not an intrinsic 2D magnet, graphene serves as a crucial component in 2D magnetic heterostructures due to its exceptional spin transport properties and weak SOC. Since its isolation in 2004 by Geim and Novoselov [19,21], graphene’s ultra-high carrier mobility, ballistic transport, and long spin coherence lengths (exceeding 100 μm at room temperature) have positioned it as an ideal spin channel for spintronic applications [52]. The linear dispersion at Dirac points and massless charge carriers enable efficient spin injection and detection when interfaced with magnetic 2D materials, making graphene indispensable for integrating intrinsic 2D ferromagnets into functional devices [53].

**Emergent Magnetism in Twisted Bilayer Graphene:** Twisted bilayer graphene at magic angles (~1.1°) exhibits emergent magnetic phenomena that complement intrinsic 2D magnets. Near three-quarters filling, these systems demonstrate spontaneous ferromagnetism alongside correlated insulating behavior, with the magnetic ground state being electrically tunable through gate voltage [54,55]. This emergence of flat-band magnetism enables twisted graphene to function not only as a spin transport medium but also as an active magnetic component in heterostructures. Additionally, twisted bilayer graphene exhibits intrinsic quantized AHE, demonstrating robust magnetic ordering comparable to intrinsic ferromagnets [54].

**Integration with 2D Magnetic Materials**: Graphene’s compatibility with vdW assembly enables seamless integration with intrinsic 2D ferromagnets including CrI_3_, F_3_GT, and CGT. In these heterostructures, graphene maintains its high mobility while acquiring spin-polarized properties through magnetic proximity effects. Key spintronic functionalities demonstrated include tunneling magnetoresistance in graphene/CrI_3_ magnetic tunnel junctions, spin filtering in graphene/F_3_GT bilayers, and SOT effects enabling current-driven magnetic switching [56,57,58,59,60,61]. The proximity-induced spin polarization in graphene can extend several nanometers from the magnetic interface, creating spin-polarized channels suitable for long-range spin transport.

**Spintronic Device Applications:** Graphene-based magnetic heterostructures have enabled breakthrough spintronic devices that leverage both materials’ advantages. Graphene/CrI_3_ tunnel junctions demonstrate gate-tunable magnetic switching with magnetoresistance ratios exceeding 100%, while graphene/F_3_GT bilayers exhibit strong magnetic proximity coupling and room temperature operation [56,58]. Recent advances have further expanded the spintronic capabilities of graphene-based systems. Karpiak et al. demonstrated seamless spin valve operation in graphene/CGT heterostructures, where proximity-induced magnetic exchange coupling enables both electrical spin injection and the spin Hall effect while retaining efficient spin transport, achieving spin diffusion lengths exceeding several micrometers [62]. Cosset-Chéneau et al. developed artificial spin-polarized graphene electrodes for magnetic tunnel junctions through proximity coupling with ferromagnetic insulators, achieving tunneling magnetoresistance and demonstrating the emergence of spin-dependent splitting (~15 meV) in graphene’s Dirac band structure, opening pathways for gate-tunable spintronic memory devices [63].

### 2.6. Mn_3_Si_2_Te_6_ (MST)

Mn_3_Si_2_Te_6_ is a vdW layered ferrimagnetic semiconductor that crystallizes in a trigonal structure with space group *P*$\bar{3}$1*c* (No. 163) in its bulk form [64]. In the monolayer limit, the structure adopts the space group *P*$\bar{3}$1*m* (No. 162), which retains only the rotational part of *P*$\bar{3}$1*c*, and both symmetries share the same point group *D_3d_* [65,66]. Each Mn atom is octahedrally coordinated by six Te atoms, forming a distorted octahedral crystal field [67]. The Mn atoms occupy the 2*c* Wyckoff positions, creating a simple hexagonal lattice, while the Si atoms are surrounded by six Te atoms.

The monolayer unit consists of five atomic layers and contains ten atoms per primitive hexagonal cell. Structurally, the bulk crystal features Mn–Te slabs separated by Si–Te layers, producing weak interlayer bonding and enabling mechanical exfoliation. The two inequivalent Mn sites (Mn1 in the intralayer honeycomb network and Mn2 in interlayer positions) give rise to ferromagnetic coupling within Mn1 planes and antiferromagnetic coupling between Mn1 and Mn2 sublattices, resulting in a net ferrimagnetic order. Magnetic ordering occurs at a T_C_ of approximately 78 K, with the magnetic moments predominantly aligned in the basal plane of the trigonal lattice [67].

Significant magnetic anisotropy arises from strong SOC due to Te orbitals hybridized with Mn spins; this proximity effect, consistent with strong Mn–Te orbital hybridization, can be readily perturbed by small direct currents. Beyond its fundamental properties, MST offers tunable magnetic ordering and spin polarization, making it attractive for spintronic devices such as magnetic memory, spin valves, and spin-based transistors [68].

Its integration into vdW heterostructures with other 2D materials, including graphene and transition-metal dichalcogenides, enhances spin injection, manipulation, and transport efficiency, while reducing power dissipation. Such heterostructures also enable functionalities like spin filtering, relevant to quantum computing and next-generation memory technologies. Cheng Tan et al. demonstrated that MST nanoflakes, as quasi-layered ferrimagnets, exhibit electrically gate-tunable colossal magnetoresistance with ultra-fast response, low current-density modulation (~5 A/cm^2^), and distinct transport characteristics compared to their bulk counterparts [69]. These unique properties also establish MST nanoflakes as an excellent platform for exploring the fundamental physics of chiral orbital moments, magnetic-field-induced band modifications, and spin–torque phenomena.

While intrinsic properties and material characteristics define the potential of 2D magnetic materials, their practical implementation requires sophisticated synthesis techniques that control layer thickness, purity, and interface quality. The following section examines the primary fabrication methods that enable the realization of high-quality 2D magnetic systems.

### 2.7. CrI_3_

CrI_3_, a chromium triiodide compound, stands as a pioneering material in the field of 2D magnetism, representing one of the first experimentally confirmed monolayer ferromagnets with intrinsic magnetic ordering down to the atomically thin limit. This layered vdW crystal exhibits Ising-type ferromagnetism with strong perpendicular magnetic anisotropy, enabling long-range magnetic order to survive thermal fluctuations at the monolayer scale, contrary to the predictions of the Mermin-Wagner theorem for isotropic 2D systems [9,10]. Structurally, CrI_3_ consists of a central Cr layer sandwiched between upper and lower I atomic layers, and the interlayer connectivity is facilitated by vdW interactions, as illustrated in Figure 4a [70].

The unique magnetic characteristics of CrI_3_ have enabled breakthrough demonstrations across multiple spintronic applications, spanning 2D magnetic tunnel junctions [59,71], topological magnetic textures including skyrmions [72,73], and emerging quantum magnetic device architectures [23,74]. The magnetic properties of CrI_3_ exhibit remarkable thickness dependence, with the bulk material displaying a T_C_ of approximately 61 K, while monolayer CrI_3_ maintains ferromagnetic order up to ~45 K [10]. CrI_3_-based heterostructures have demonstrated exceptional spintronic performance, with tunneling magnetoresistance exceeding 19,000% in CrI_3_/graphene/CrI_3_ spin-filter devices and magnetoresistance ratios up to 10,000% in field-effect configurations [59,71].

**Figure 5:**
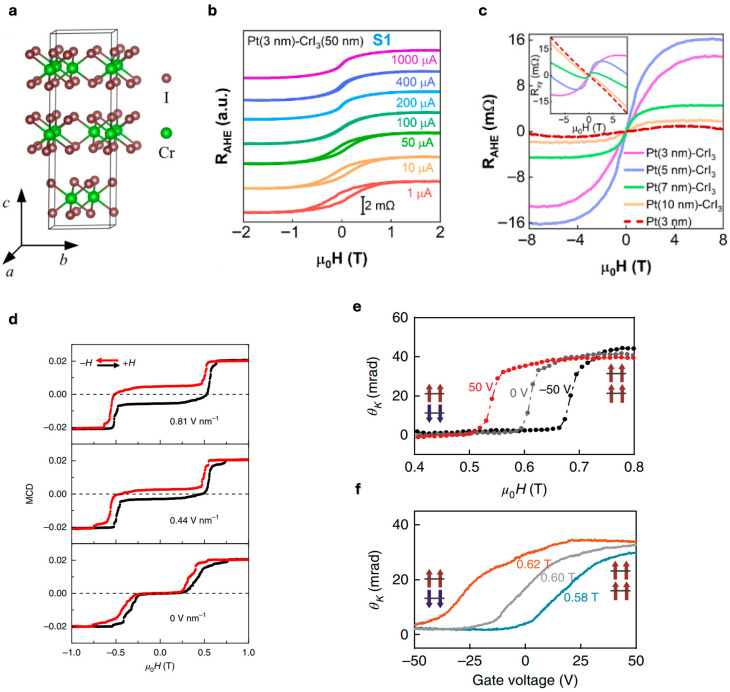
Crystal structure and magnetism of CrI_3_ single crystal. (**a**) Crystal structure of CrI_3_ bulk showing layered vdW architecture. Reprinted with permission from ref. [70]. (**b**) Current-dependent R_AHE_-H curves of Pt (3 nm)/CrI_3_ (50 nm) at 2 K, demonstrating systematic decrease in coercive field with increasing current from 1 to 1000 μA. Reprinted with permission from ref. [75]. (**c**) Thickness-dependent R_AHE_-H curves of Pt films with varying thicknesses and CrI_3_ films (constant 60 nm thickness), showing progressive weakening of anomalous Hall signal with increasing Pt thickness. The inset illustrates the R_AHE_-H curve obtained at 2 K. Reprinted with permission from ref. [75]. (**d**) MCD signal of bilayer CrI_3_ as a function of magnetic field under electric fields of 0, 0.44, and 0.81 V nm^−1^ at 4 K. The critical field systematically decreases from ~0.65 T to ~0.35 T with increasing electric field, demonstrating voltage-controlled magnetoelectric switching. Reprinted with permission from ref. [76]. (**e**) Gate voltage dependence of critical magnetic field showing systematic tuning from 0.5 T to 0.7 T. Reprinted with permission from ref. [74]. (**f**) Gate-induced AFM-to-FM transitions at fixed magnetic fields 0.58 T, 0.60 T, and 0.62 T, demonstrating complete electrical control over magnetic ordering with decreasing gate voltage threshold as magnetic field increases. Reprinted with permission from ref. [74].

Recent studies by Zheng et al. (2024) demonstrated that Pt thin films deposited on CrI_3_ nanoflakes exhibit robust AHE, indicating the induction of spontaneous magnetization in Pt through magnetic proximity effects [75]. The AHE serves as a direct probe of the proximity-induced magnetization in Pt/CrI_3_ heterostructures. As demonstrated in Figure 5b, current-dependent measurements reveal that both the coercive field and saturation resistance systematically decrease with increasing applied current from 1 to 1000 μA, with the coercive field becoming negligible at 100 μA. Thickness-dependent studies shown in Figure 5c demonstrate that increasing Pt thickness leads to progressive reduction in the anomalous Hall signal and eventual disappearance of proximity-induced magnetization, confirming that the magnetic proximity effect is predominantly an interfacial phenomenon.

CrI_3_ demonstrates exceptional voltage-controlled magnetic properties, making it a prime candidate for electrically tunable spintronic devices. Jiang et al. (2018) achieved reversible magnetoelectric switching in bilayer CrI_3_ heterostructures by implementing graphene electrodes and hBN as the gate dielectric layer [76]. As demonstrated in Figure 5d, MCD signal measurements at different electric field strengths (0.44 and 0.81 V/nm) reveal systematic control over the metamagnetic transition, with the critical field decreasing from ~0.65 T to ~0.35 T as electric field increases. Their work demonstrated that applying electric fields below 0.2 V/nm preserves the ferromagnetic state, while fields exceeding 0.7 V/nm induce a transition to antiferromagnetic coupling enabling voltage-controlled magnetic phase transitions.

Huang et al. (2018) further demonstrated comprehensive gate-voltage control of magnetic phase transitions in bilayer CrI_3_ [74]. As shown in Figure 5e, the critical magnetic field for the metamagnetic transition can be systematically tuned by gate voltage, with the transition shifting from ~0.5 T at +50 V to ~0.7 T at −50 V, demonstrating bidirectional control over the magnetic phase boundary. Complementary measurements in Figure 5f reveal gate-induced magnetic transitions at fixed magnetic fields, where the MOKE signal transitions from near-zero (AFM state) to saturated values (FM state) as gate voltage increases past critical thresholds. At selected magnetic fields of μ_0_H = 0.58 T (blue), 0.60 T (grey), and 0.62 T (orange), the required gate voltage for AFM-to-FM switching systematically decreases with increasing magnetic field, enabling complete electrical control over magnetic ordering.

To facilitate systematic comparison across these material systems, Table 1 summarizes the key intrinsic properties of the 2D magnetic materials discussed above. This comparative overview highlights the distinct characteristics that position each material for specific spintronic applications—ranging from cryogenic quantum devices to near room temperature memory technologies. The table emphasizes critical parameters including T_C_, magnetic anisotropy orientation, structural characteristics, and electrical tunability, which collectively govern their integration pathways into functional devices.

## 3. Synthesis Techniques for 2D Magnetic Materials

The controlled synthesis of high-quality 2D magnetic materials represents a cornerstone for advancing next-generation spintronic devices. Several fabrication methods have been developed to yield monolayer and few-layer magnetic systems, each offering distinct advantages in terms of crystal quality, scalability, and interface control. Among the most widely adopted synthesis strategies are chemical vapor deposition (CVD), micromechanical exfoliation with dry and wet transfer techniques, and molecular beam epitaxy (MBE). Each method plays a unique role in enabling tunable magnetism and precise interface engineering in vdW heterostructures—essential for unlocking new physical phenomena and functionalities in 2D spintronic platforms.

### 3.1. Chemical Vapor Deposition

Chemical Vapor Deposition (CVD) is one of the most widely adopted methods for synthesizing high-quality, large-area thin films of 2D materials, including magnetic vdW crystals such as F_3_GT and CrI_3_ [89,90]. In this process, vapor-phase precursors are introduced into a reaction chamber, where they undergo controlled chemical transformations to deposit crystalline thin layers on a heated substrate [91]. Parameters such as substrate temperature, gas flow rates, and ambient pressure can be finely tuned to control the thickness, crystallinity and composition of the resulting films.

In the context of 2D magnets, modifications of CVD protocols enable the formation of ferromagnetic layers with atomically sharp interfaces and robust magnetic order. For instance, CrI_3_ can be synthesized by evaporating CrI_3_ powder in a carrier gas, depositing ultrathin ferromagnetic layers on substrates with magnetic order persisting down to the monolayer limit [92]. Such monolayers exhibit excellent spin-polarized behavior, making them ideal candidates for integration into spintronic devices.

The scalability of CVD and its compatibility with various substrates make it an attractive technique for wafer-scale production [93,94]. However, substrate choice and interface quality are critical to maintaining magnetic integrity, and current research aims to optimize lattice matching and growth conditions for emerging 2D magnets [80,95].

### 3.2. Micromechanical Exfoliation and Transfer Techniques

Micromechanical exfoliation remains a foundational approach for producing high-purity, monolayer or few-layer 2D magnetic crystals. Based on the weak interlayer vdW bonding in layered materials, this method enables physical delamination of layers using either dry or wet transfer protocols [96]. The iconic “Scotch tape” method is particularly notable for isolating monolayer graphene and has since been adapted to obtain pristine flakes of magnetic crystals such as CrI_3_, CrCl_3_, and F_3_GT [2,97].

A typical procedure involves pressing adhesive tape onto a bulk crystal (e.g., graphite or CrI_3_), peeling it away to lift thin flakes, and transferring them onto a target substrate—usually SiO_2_/Si or sapphire—by gently pressing and slowly removing the tape [1,98]. The exfoliated flakes adhere to the substrate through vdW forces, retaining their structural and magnetic integrity [92,99]. This method, while not scalable, remains indispensable for fundamental studies requiring atomically clean and defect-free 2D magnets.

Numerous 2D magnetic materials have been isolated via this approach, including CrI_3_, CrCl_3_, CrSiTe, CrGeTe_3_, Fe_3_GeTe_2_, FePS_3_, MnPS_3_, and NiPS_3_ [81,97,100]. These exfoliated nanoflakes form the basis for constructing vdW heterostructures used in spin filters, tunnel junctions, and quantum sensors. Following exfoliation, the flakes are transferred onto desired substrates using either dry or wet transfer methods, each suited for different material types and device architectures.

#### 3.2.1. Dry Transfer

The dry transfer technique involves mechanical delamination of 2D layers from bulk crystals using adhesive tapes [101,102]. This technique, notably used to exfoliate graphene from graphite, can be applied to other 2D ferromagnetic materials [103,104]. The process involves placing a bulk crystal onto adhesive tape and using this to remove thin flakes of the material. These flakes are then transferred onto a substrate, preferably SiO_2_/Si or sapphire, by placing the tape on the substrate and slowly peeling it off [105].

The dry transfer method employs a polymeric stamp to pick up 2D material and transfer it onto a substrate without using any soluble media. This technique enables control over thin layers, which are needed to create heterostructures for spintronics. The advantages of dry transfer include ease of processing and creation of high-quality, clean 2D layers free of contamination [58,106,107,108]. The technique is well-suited for materials that are easily degraded through chemical processes or solvents. For instance, monolayers of F_3_GT and CGT have been synthesized using dry transfer methods, facilitating investigation of material properties without the influence of chemical residues.

However, dry transfer has limitations. Although it is possible to obtain monolayers with relatively high yields, the yield and control over layer thickness vary, and the process is relatively time-consuming. Furthermore, the positioning of flakes directly onto the substrate can be imprecise, which is important for achieving well-defined interfaces in heterostructures.

#### 3.2.2. Wet Transfer

Wet transfer relies on solvent-mediated delamination, enabling the deposition of exfoliated layers onto substrates through liquid-phase processing. This method generally uses solvents or intercalation agents to disrupt the vdW interactions between layers to facilitate their separation [109]. The exfoliated layers are then dispersed in a solvent, and the dispersion is coated onto the substrate via techniques such as drop casting, spin coating, or filtration.

Wet transfer can be accomplished through liquid-phase exfoliation, in which bulk crystals are suspended in a solvent and ultrasonicated to produce 2D flakes [61,110]. The suspension is then filtered or centrifuged to obtain thin layers and subsequently coated onto a substrate. This manufacturing technique is ideal for producing large quantities of 2D material, making it versatile for mass production.

Another wet transfer process involves transferring 2D material onto a water-soluble layer. The layer can be dissolved in water, allowing the material to float and then be transferred to another substrate. This technique is applied broadly in synthesizing high-quality, defect-free heterostructures for 2D materials. Wet transfer has proved effective in preparing monolayers and few-layered structures of numerous 2D ferromagnetic compounds, including CrTe_2_ and F_5_GT. This technique enables better control of thickness and lateral size of the flakes compared to dry transfer, making it preferable for preparing large-area films and devices.

Wet transfer has certain drawbacks despite its advantages. The process may result in chemical residues and impurities from solvents and intercalation agents in the composite material [108,110]. Additionally, transferred layers can develop wrinkles, cracks, and other structural imperfections that may adversely affect device functionality.

### 3.3. Molecular Beam Epitaxy

Molecular beam epitaxy (MBE) is a highly controlled, ultra-high-vacuum (UHV)-based technique widely employed for the layer-by-layer synthesis of high-purity and atomically sharp 2D materials [111,112]. In this process, molecular or atomic beams of source materials are thermally evaporated and directed toward a heated substrate, where epitaxial thin films grow under carefully regulated conditions. The in situ monitoring of growth using tools such as reflection high-energy electron diffraction (RHEED) allows for precise control over film thickness, crystallinity, and composition [113,114].

MBE is especially advantageous for fabricating vdW magnetic heterostructures with clean and well-defined interfaces, which are essential for probing spin-dependent phenomena such as exchange bias, SOC, and interlayer magnetic proximity effects. This makes MBE a preferred method for fundamental investigations into quantum magnetism and 2D spintronics.

High-quality films of magnetic transition metal dichalcogenides, including F_5_GT, Cr_2_Te_3_, and VSe_2_, have been synthesized via MBE with a controllable layer number and stoichiometry [43,112]. These epitaxial films exhibit enhanced magnetic ordering, tunable Curie temperatures, and clean vdW interfaces suitable for device integration. Additionally, MBE facilitates the fabrication of hybrid heterostructures combining magnetic layers with topological insulators, semiconductors, or superconductors—paving the way toward realizing exotic quasiparticles such as Majorana fermions for quantum computation [112,115].

Despite its exceptional precision, MBE is limited by its high operational cost and low throughput, making it less suitable for large-scale manufacturing. Nonetheless, it remains a cornerstone technique for the exploratory growth of novel 2D magnetic systems and for elucidating interface-driven magnetic phenomena with an atomic resolution.

The ability to tailor synthesis conditions directly impacts the resulting electronic and magnetic structures, which in turn determine spintronic performance. Having established fabrication methods that enable high-quality 2D magnetic materials, we now examine how their electronic band structures govern magnetic behavior and transport phenomena.

## 4. Electronic and Magnetic Structure

The electronic structure of a material—characterized by the arrangement and energy distribution of electrons across bands—directly influences its magnetic behavior, especially in low-dimensional systems [25]. Complementarily, the magnetic structure pertains to the orientation and alignment of magnetic moments within the crystal lattice, which governs the material’s overall magnetic ordering [116]. In 2D magnetic materials, these two structures are intricately linked, jointly dictating the electrical conductivity, spin polarization, and magnetic response at the nanoscale [117].

For instance, CGT possesses a layered hexagonal structure composed of Cr atoms sandwiched between Ge and Te layers. It exhibits a direct bandgap whose magnitude can be modulated via external strain or chemical doping, making it highly responsive to external perturbations. Moreover, SOC plays a pivotal role in determining its magnetocrystalline anisotropy and long-range ferromagnetic order [89]. In contrast, F_3_GT features metallic electronic properties with distinct interlayer bonding characteristics. Its magnetism arises from localized Fe moments, and strong electron-electron interactions promote robust ferromagnetic ordering even at elevated temperatures, which is crucial for device-grade thermal stability [118].

In spintronic systems, the coupling between a material’s band structure and its magnetic properties plays a fundamental role in device functionality. In magnetic semiconductors, spin-resolved band structures often feature spin-splitting near the Fermi level, giving rise to spin polarization—an essential mechanism for realizing spin-filters, spin-injectors, and magnetic tunneling junctions [119]. The magnitude and directionality of SOC further modulate the band structure and magnetic anisotropy, especially in 2D materials composed of heavy elements such as Te or Bi, where relativistic effects are pronounced [109].

For example, F_3_GT, a layered metallic ferromagnet, derives its magnetic moment primarily from localized Fe 3d orbitals. Its band structure remains gapless but strongly spin-polarized, and exhibits pronounced SOC-induced anisotropy. These characteristics make F_3_GT highly responsive to external electric gating and strain, allowing for tunable magnetic switching, which is indispensable for reconfigurable spin logic and memory applications [36]. Hence, understanding and engineering the interplay between electronic dispersion and magnetic alignment is central to the rational design of vdW spintronic heterostructures.

The atomic-scale electronic structure and bonding configuration play a pivotal role in the formation, stability, and manipulation of magnetic domains in 2D materials. In vdW magnetic systems, domain behavior can be precisely tailored through the control of stacking orientation, application of interlayer pressure, or electrostatic gating—all of which influence interfacial charge distribution and exchange interactions [120]. Modifications at the interface can induce orbital hybridization and alter exchange coupling strength, directly impacting magnetization switching thresholds and spin dynamics.

Moreover, intrinsic defects, grain boundaries, and local strain fields within the crystal lattice can act as pinning centers for domain walls or nucleation sites for exotic spin textures such as skyrmions [13,121]. These localized perturbations modulate both the static and dynamic magnetic responses, emphasizing the need for atomic-level electronic control in the design of efficient, robust spintronic architectures. Thus, a comprehensive understanding of the interplay between electronic bonding and magnetic topology is essential for advancing spintronic logic, memory, and neuromorphic functionalities.

### 4.1. Electronic Band Structures

The electronic band structure provides the foundation for understanding how electrons behave in 2D magnetic materials and determines their transport and magnetic properties. Several key features distinguish 2D magnetic systems from their bulk counterparts and enable novel functionalities.

#### 4.1.1. Dirac-like and Weyl Fermions

In some 2D magnetic materials, electronic states resemble massless Dirac or Weyl fermions similar to those in graphene [122]. However, the presence of magnetism introduces new and interesting features to these states. For example, in CrI_3_, the interplay between magnetic order and Dirac-like fermions results in spin-polarized bands with different energy levels for spin-up and spin-down electrons. This opens pathways for new quantum states with potential applications in quantum computing and spintronics [116].

#### 4.1.2. Flat Bands

In certain 2D magnetic materials, electronic band structures exhibit nearly dispersionless (flat) bands across momentum space, resulting in a high density of states at specific energy levels [27]. These flat bands significantly enhance electron-electron interactions, giving rise to emergent correlated phenomena such as magnetism, Mott insulating behavior, and unconventional superconductivity. A prominent example is twisted bilayer graphene, where moiré-induced flat bands at “magic angles” lead to correlated insulating and superconducting ground states [123,124]. The presence of flat bands in 2D magnetic systems opens new avenues for engineering exotic quantum phases, making them compelling platforms for strongly correlated spintronic devices.

#### 4.1.3. Spin-Polarized Bands

In 2D magnetic materials, spin polarization emerges when electronic bands split according to spin orientation, yielding distinct energy levels for spin-up and spin-down carriers. This intrinsic band asymmetry forms the foundational mechanism of spintronic functionality, enabling control over spin currents rather than mere charge transport [125]. The degree of spin polarization is governed by the strength of intralayer exchange interactions and SOC, both of which can be engineered through external stimuli or heterostructure design. Materials such as CGT, F_3_GT, and CrI_3_ have been experimentally shown to exhibit strong spin polarization, making them ideal for spin-filtering and spin-injection applications in spintronics [9,126].

### 4.2. Density of States

The describes the number of available electronic states at each energy level and plays a critical role in determining how electrons occupy bands in a material. In 2D systems, the DOS strongly influences electrical conductivity, magnetoresistance, and spin transport characteristics. Sharp peaks in the DOS—known as Van Hove singularities—can enhance electronic correlations, thereby amplifying phenomena such as magnetism, charge density waves, or unconventional superconductivity [127]. Furthermore, the DOS profile in 2D materials can be dynamically engineered through strain, layer stacking, or external gating, enabling fine control over the material’s quantum response for targeted spintronic applications.

#### 4.2.1. Van Hove Singularities

Van Hove singularities represent critical points in the DOS where the band structure features saddle points in momentum space, leading to pronounced peaks in the DOS. These singularities enhance electronic correlations, significantly influencing both magnetic and electronic ground states [128]. In 2D magnetic materials, particularly transition metal dichalcogenides, Van Hove singularities have been shown to mediate competing ferromagnetic and antiferromagnetic interactions, thereby governing the emergence of long-range magnetic order or other correlated phenomena such as Mott insulating states and charge density waves [129]. Their tunability via strain or twist angle engineering makes them a versatile design parameter for next-generation spintronic devices.

#### 4.2.2. Spin-Dependent Density of States

Spin-dependent density of states is a hallmark feature of magnetic 2D materials, wherein the DOS for spin-up and spin-down electrons differs due to spin splitting. This phenomenon arises primarily from strong intralayer exchange interactions and SOC, which lift the degeneracy of electronic states based on spin orientation [117]. Such asymmetry in spin-resolved DOS is fundamental to generating spin-polarized currents—an essential prerequisite for the operation of spintronic devices [13,130]. A notable example is CrI_3_, where the spin-dependent DOS underlies its giant magnetoresistance behavior, making it a viable candidate for application in magnetic sensors, non-volatile memory elements, and spin-filtering junctions.

#### 4.2.3. Magnetic Proximity Effects in Graphene

Although pristine graphene is intrinsically nonmagnetic, its electronic and spin properties can be profoundly modified through magnetic proximity coupling when brought into contact with 2D ferromagnetic materials. This interfacial interaction induces spin splitting in graphene’s Dirac bands, resulting in a spin-polarized density of states without the need for magnetic doping or external fields [104,108,131]. Such proximity effects have been experimentally demonstrated in heterostructures involving graphene and magnetic layers like CGT or F_3_GT, where spin-dependent band modulation enables control over spin injection and transport.

This integration maintains graphene’s superior carrier mobility while imparting magnetic functionality, making it a powerful platform for spintronic components such as spin filters, valves, and gate-tunable magnetic tunnel junctions [132]. The proximity-induced magnetism in graphene represents a prime example of how interface engineering in vdW heterostructures can create new functionalities that neither constituent material possesses individually.

The electronic and magnetic band structures of 2D materials—ranging from flat bands and spin-polarized density of states to proximity-induced magnetism—serve as a foundational framework for modern spintronic technologies. These features not only govern how spin currents are generated and manipulated but also determine the transport behavior in complex heterostructures. The ability to tailor these properties through external fields, strain engineering, or interface design enables the realization of highly tunable and multifunctional devices. Building upon this understanding, the following section explores how such engineered quantum states translate into emergent spin transport phenomena—most notably, tunneling magnetoresistance, the QAHE, and exchange bias—underpinning the next generation of low-dimensional spintronic applications.

## 5. Transport Studies in 2D Spintronic Device Applications

Transport phenomena in 2D magnetic materials and their vdW heterostructures offer profound insights into charge and spin carrier dynamics. These studies reveal the key mechanisms underpinning conductivity, magnetoresistance, spin polarization, and topological effects—fundamentals for developing energy-efficient, high-speed spintronic devices. Multiple transport behaviors, such as magnetic anisotropy, giant magnetoresistance (GMR), the quantum anomalous Hall effect (QAHE), spin–orbit torque (SOT), exchange bias (EB), skyrmions, and tunneling magnetoresistance (TMR), have been observed in 2D systems. These phenomena are intimately tied to the reduced dimensionality, interfacial coupling, and spin–orbit interactions present in these atomically thin materials.

### 5.1. Magnetic Anisotropy

Magnetic anisotropy—the directional dependence of a material’s magnetic properties—is essential for stabilizing long-range ferromagnetic order in 2D crystals, which otherwise would be suppressed due to thermal fluctuations, as predicted by the Mermin-Wagner theorem [74,133]. Out-of-plane anisotropy is particularly critical in 2D magnets to achieve robust magnetic phases at finite temperatures. Materials such as F_3_GT and CrI_3_ exhibit significant perpendicular magnetic anisotropy due to strong SOC and symmetry breaking at the atomic scale [134,135].

This anisotropy enables persistent spin alignment in few-layer systems, making them suitable for spintronic memory applications. Moreover, external electric fields have been shown to dynamically modulate magnetic anisotropy in vdW heterostructures, enabling tunable spin logic architecture [136,137]. Strain engineering offers an additional route to tailor the magnitude and direction of anisotropy, facilitating the design of flexible and reprogrammable spintronic devices.

### 5.2. Giant Magnetoresistance (GMR)

The phenomenon of giant magnetoresistance was first observed in Fe/Cr multilayers by Baibich et al. (1988) and Binasch et al. (1989), introducing the electron spin as a practical element in electronics and earning the 2007 Nobel Prize in Physics [138,139]. In conventional GMR trilayers, two ferromagnetic layers are separated by a non-magnetic spacer, resulting in sharply contrasting resistances between parallel (low resistance) and antiparallel (high resistance) magnetic alignments—a principle instrumental in magnetoresistive random-access memory (MRAM) and magnetic sensors.

The GMR concept has been successfully realized in vdW heterostructures, where atomically clean interfaces between materials such as F_3_GT and graphene facilitate exceptional spin transport with minimal scattering, leading to remarkably high magnetoresistance performance [57]. A landmark study reported a room temperature GMR exceeding ~9400% in F_3_GT/graphene heterostructures, maintaining high magnetoresistance across a temperature range from 4 K to ambient conditions—a behavior attributed to spin-dependent orbital coupling at the F_3_GT/graphene interface [140].

First-principles theoretical work supports the potential of vdW GMR devices: in a current-perpendicular-to-plane junction composed of F_3_GT electrodes and PtTe_2_ or PdTe_2_ spacers, GMR ratios of ~2000% were predicted alongside low-resistance-area products (< 0.3 Ω·μm^2^) and anisotropic magnetoresistance (~20%)—outlining a path to room temperature operability and high device efficiency [140].

Moreover, F_3_GT/graphite/F_3_GT trilayer devices demonstrated a rare antisymmetric magnetoresistance effect, showing three distinct resistance states due to spin filtering and quantum coherence at the vdW interfaces—deviating from conventional two-state GMR behavior [57]. These experimental and computational advances highlight that weak interlayer coupling and pristine vdW interfaces significantly extend spin coherence and enable tuning of magnetoresistance via external stimuli (gate voltage, strain, doping), positioning vdW-based GMR platforms as promising candidates for scalable, low-power, and reconfigurable spintronic devices.

### 5.3. Quantum Anomalous Hall Effect (QAHE)

The quantum anomalous Hall effect is a quantum transport phenomenon in which a system exhibits quantized Hall conductance without an external magnetic field. This effect stems from intrinsic SOC and spontaneous magnetization, enabling dissipationless edge current transport, making it particularly attractive for low-power spintronic and topological quantum devices.

In 2D systems, achieving QAHE relies on the fine interplay between magnetic ordering and topologically non-trivial band structures. The earliest experimental realization of QAHE was demonstrated in Cr- or V-doped (Bi,Sb)_2_Te_3_ thin films, confirming theoretical predictions and paving the way for magnetic topological insulators [52]. Since then, considerable efforts have been made to identify and engineer intrinsic 2D magnetic materials capable of supporting QAHE at higher temperatures.

VdW magnetic materials, such as F_3_GT and MnBi_2_Te_4_, have emerged as promising candidates due to their intrinsic out-of-plane ferromagnetism and strong SOC [141,142]. MnBi_2_Te_4_, in particular, serves as a layered antiferromagnetic topological insulator capable of hosting QAHE through field-tuned magnetic configurations. Such materials, when combined with vdW heterostructures, provide atomically sharp interfaces and minimal defect scattering—conditions favorable for robust edge states and topological protection.

Furthermore, proximity-induced magnetism in non-magnetic topological layers such as graphene and WTe_2_ has been shown to induce QAHE-like behavior when interfaced with 2D ferromagnets, enabling voltage-tunable band topology [143,144]. This has sparked the design of gate-tunable QAHE devices and modular heterostructures for reconfigurable topological electronics. The realization of a room temperature QAHE in 2D vdW heterostructures would mark a major leap toward practical topological spintronic circuits.

### 5.4. Spin–Orbit Torque (SOT)

SOT represents a pivotal mechanism for manipulating magnetization in spintronic devices by leveraging SOC effects. In systems with broken inversion symmetry or strong SOC—such as heavy metals, topological insulators, or transition metal dichalcogenides—a charge current can generate a transverse spin current via either the spin Hall effect or the Rashba–Edelstein effect, which then exerts torque on an adjacent ferromagnetic layer, switching its magnetization without the need for external magnetic fields [145,146].

In 2D vdW heterostructures, the SOT effect has gained remarkable traction due to the atomically sharp interfaces, reduced dimensionality, and the strong SOC of many 2D materials. Particularly, 2D transition metal dichalcogenides such as WTe_2_, MoTe_2_, and NbSe_2_ have emerged as efficient SOT generators when interfaced with ferromagnetic materials like F_3_GT and CGT [147,148]. For instance, WTe_2_/F_3_GT heterostructures have demonstrated robust SOT-induced magnetization switching at low current densities and without an external magnetic field, thanks to the pronounced spin Hall effect and symmetry breaking in WTe_2_ [149].

The symmetry and orientation of the transition metal dichalcogenide layer play a significant role in determining the nature of the SOT. In WTe_2_, for example, current-induced torques show a strong angular dependence linked to its low-symmetry crystal structure, making it a model system for symmetry-driven spin dynamics [149]. One of the most significant advantages of employing SOT in 2D systems is their ability to facilitate field-free, energy-efficient magnetization switching, which is essential for scalable memory technologies such as SOT-MRAM. These devices offer non-volatility, high endurance, and faster switching times compared to traditional spin-transfer torque MRAM, especially when integrated with 2D materials exhibiting strong interfacial SOC [145].

Furthermore, gate tuning of SOT has been demonstrated in 2D vdW heterostructures, enabling dynamic control over spin transport and torque generation. Gate-modulated SOTs open pathways to programmable spin logic architectures and multifunctional memory systems [150]. Proximity effects between nonmagnetic 2D layers with high SOC and adjacent ferromagnetic layers further enhance the magnitude and tunability of SOT, contributing to the reconfigurability and robustness of spintronic platforms.

### 5.5. Exchange Bias (EB)

Exchange bias is a critical magnetic phenomenon that manifests as a horizontal shift in the hysteresis loop of a ferromagnet due to interfacial coupling with an antiferromagnet. This unidirectional anisotropy stabilizes magnetization, which is essential for non-volatile magnetic memory and spintronic applications [151,152].

In 2D vdW heterostructures, EB can be engineered by vertically stacking ferromagnet and antiferromagnet layers with atomically clean interfaces. For instance, Albarakati et al. (2022) demonstrated gate-tunable EB in FePS_3_–F_5_GT vdW heterostructures, where electrostatic gating via a solid proton conductor modulated the EB field up to ~23% of the coercive field, with a blocking temperature near 60 K [39]. Similarly, Ye et al. (2023) achieved laser-induced control of EB in FePSe_3_/F_3_GT by dynamically compressing the interlayer spacing, increasing the EB field from ~30 mT to 111 mT and extending the blocking temperature to ~110 K [153].

Another promising example is the CrSBr/F_3_GT system, where interfacial anisotropy contrast induced tilted in-plane EB and enabled deterministic field-free SOT switching—an essential requirement for next-generation logic devices [154]. Additionally, Sharma et al. (2024) reported a record-high gate-controlled EB field (~1.4 kOe) in a F_3_GT/O_x_-F_3_GT/hBN stack, showing voltage-tunable magnetization switching at low energy costs [155].

Theoretical studies predict that electrically tunable EB can emerge in heterostructures such as MnBi_2_Te_4_/CGT, suggesting a pathway toward integrating topological quantum phenomena with magnetic order [156]. These results collectively emphasize the unique advantages of 2D vdW materials for EB control, enabling dynamic, energy-efficient, and field-free operation for spin-based logic and memory architectures.

### 5.6. Tunneling Magnetoresistance (TMR)

Tunneling magnetoresistance is a quantum mechanical phenomenon that arises in magnetic tunnel junctions, where two ferromagnetic layers are separated by an ultrathin insulating barrier. The electrical resistance of the junction depends sensitively on the relative alignment of the magnetizations in the ferromagnetic electrodes: it is significantly lower when the magnetizations are parallel and higher when they are antiparallel, a behavior attributed to spin-dependent electron tunneling across the barrier [157]. Compared to GMR, TMR exhibits a larger resistance change and is foundational in contemporary spintronic devices, including MRAM and spin-transfer torque-based technologies [157].

The integration of 2D magnetic materials into magnetic tunnel junction architecture has introduced a transformative paradigm for controlling spin-dependent transport at the atomic scale. Due to their atomically thin nature, reduced dimensionality, and defect-free vdW interfaces, 2D magnets offer precise tunability of interfacial spin polarization and tunneling barrier characteristics [56,59]. For instance, heterostructures using hexagonal boron nitride or semiconducting transition metal dichalcogenides like WSe_2_ as tunnel barriers have shown enhanced TMR ratios and low-power switching characteristics [59].

Several 2D ferromagnetic materials, including F_3_GT and CrI_3_, have emerged as strong candidates for TMR-based spintronic applications. These materials exhibit high Curie temperatures, robust magnetic ordering in few-layer forms, and compatibility with vdW integration techniques. Notably, vdW heterostructures such as F_3_GT/WSe_2_/F_3_GT have demonstrated room temperature tunable TMR behavior through electrostatic gating [158,159]. Additionally, spin-filtering-based heterostructures such as CrI_3_/hBN/graphene show giant TMR effects due to spin-polarized tunneling and magnetic state control [59].

As the field advances, optimizing the choice of 2D magnetic electrodes and tunnel barriers will be critical for enhancing the TMR response and device scalability. The unique properties of 2D magnets—such as perpendicular magnetic anisotropy, high spin coherence, and reduced spin scattering—underscore their promise for next-generation ultrathin spintronic architectures.

### 5.7. Skyrmions

Magnetic skyrmions are topologically protected spin textures characterized by a whirling configuration of spins that behave like quasiparticles. Their intrinsic stability against external perturbations, combined with their nanoscale size and ultralow depinning current, makes them highly attractive for ultradense, non-volatile, and energy-efficient spintronic memory and logic applications [5,160].

In 2D systems, skyrmions can be stabilized through the delicate interplay of several interactions—primarily the Dzyaloshinskii–Moriya interaction (DMI), perpendicular magnetic anisotropy, dipolar interactions, and external magnetic fields. The broken inversion symmetry at interfaces or in non-centrosymmetric crystal structures facilitates the emergence of DMI, which is crucial for the formation and stabilization of Néel-type skyrmions in vdW materials [161,162,163].

Experimental studies have demonstrated the formation of skyrmions in layered vdW ferromagnets such as F_3_GT and CGT under low-temperature conditions. These skyrmions are often stabilized through interfacial engineering, such as coupling with heavy metals to induce interfacial DMI, or via proximity effects in vdW heterostructures [164,165]. In particular, F_3_GT has shown promising behavior due to its strong perpendicular magnetic anisotropy, metallic conductivity, and tunable T_C_, making it a favorable host for skyrmionic states [163,165].

Moreover, 2D skyrmions exhibit unique gate-tunability, allowing control of their size, density, and stability through electrostatic modulation. Optical techniques such as light-induced heating or femtosecond laser pulses have also been employed to manipulate skyrmion creation and motion, enabling new paradigms for opto-spintronics and neuromorphic computing [166,167]. These advancements position 2D skyrmions as key elements in the future of reconfigurable, high-speed, and low-energy spintronic devices. The ability to stabilize, control, and manipulate skyrmions in vdW heterostructures presents a transformative route toward ultrathin logic and memory platforms.

## 6. Conclusions and Outlook

This review has examined the synthesis, intrinsic properties, and electronic transport phenomena of 2D magnetic vdW heterostructures, establishing their potential as a transformative platform for spintronic technologies. Key achievements include giant magnetoresistance exceeding 9400% in F_3_GT/graphene systems, room temperature ferromagnetism in CrTe_2_ and F_5_GT (T_C_ up to 310 K), an electrically tunable EB with blocking temperatures approaching 110 K, and demonstrations of QAHE, SOT switching, and controllable skyrmion formation. These advances showcase the unique advantages of atomically precise vdW interfaces, reduced dimensionality effects, and unprecedented electrical tunability in 2D magnetic heterostructures.

### 6.1. Critical Challenges for 2D Magnetic vdW Heterostructures

1. RoomTemperature Quantum Transport in vdW Systems. While F_5_GT and CrTe_2_ exhibit ferromagnetism above 300 K, quantum transport phenomena—including GMR, EB, QAHE, SOT, and TMR—remain largely confined to cryogenic temperatures. Achieving room temperature operation requires (i) enhanced magnetic exchange through vdW interface coupling; (ii) understanding temperature-dependent SOC evolution in layered magnets; and (iii) exploiting flat bands in twisted heterostructures to stabilize correlation-driven magnetism—fundamental condensed matter physics questions with direct technological implications.
2. Scalable Synthesis of 2D Magnetic Heterostructures. Current demonstrations use mechanically exfoliated flakes (<100 μm). Wafer-scale integration demands (i) CVD/MBE protocols for monolayer uniformity and controlled stoichiometry; (ii) eliminating defects that degrade magnetoresistance in vdW junctions; (iii) air-stable passivation preserving magnetic properties; and (iv) deterministic stacking for precise interlayer registry control.
3. Protonic Gating and Exchange Bias Control in vdW Heterostructures. Solid-state protonic gates have enabled T_C_ enhancement (F_3_GT: 220→300 K) and FM↔AFM transitions (F_5_GT), revealing rich physics of ionic intercalation effects on magnetic ordering:
  (i) Protonic Intercalation in vdW Gaps: Understanding proton diffusion pathways, intercalation-induced effects on magnetic exchange interactions, cycling endurance limits, and kinetics constraining switching speeds covers fundamental questions in electrochemistry and magnetism coupling.
  (ii) EB at vdW Interfaces: EB in AFM/FM heterostructures shows remarkable tunability (30→111 mT via compression in FePSe_3_/F_3_GT; 1.4 kOe via gating in F_3_GT/Ox-F_3_GT). Predictive understanding requires quantifying interfacial anisotropy–EB relationships from first principles, understanding charge-transfer effects on uncompensated spins, establishing thickness scaling laws for blocking temperatures, and achieving an electrically programmable EB directionality.
  (iii) Stacking-Angle Effects on Interlayer Coupling: The crystal orientation in vdW heterostructures modulates exchange interactions through moiré periodicity and orbital overlap. Critical condensed matter physics questions include systematic mapping of EB versus twist angles, understanding competing FM/AFM coupling mechanisms, and exploiting crystallographic symmetry for voltage-controlled magnetism without external fields.
4. VdW Heterostructure Integration with Electronics Practical spintronic devices require
  (i) Low-resistance contact to 2D ferromagnets without degrading vdW interfaces.
  (ii) Selective-area growth or transfer onto patterned substrates.
  (iii) Multi-terminal spin logic with cascadability.
  (iv) Thermal budget compatibility (<400 °C) while maintaining magnetic functionality.

### 6.2. Outlook: Fundamental Physics and Technological Promise

Addressing these challenges advances both our fundamental understanding and technological capabilities. From a condensed matter physics perspective, 2D magnetic vdW heterostructures provide unique platforms for exploring quantum phase transitions in reduced dimensions, interplay between topology and magnetism, emergent phenomena at engineered interfaces, and voltage control of correlated electron systems.

Technologically, voltage-controlled magnetic tunnel junctions in vdW heterostructures promise sub-attojoule switching for non-volatile memory. Protonic-gate-tunable devices enable analog computation with ultralow-power dissipation. Room temperature QAHE in vdW systems would revolutionize interconnects, while reconfigurable exchange bias enables programmable spin logic.

The atomically precise control in 2D magnetic vdW heterostructures—through protonic gating, twist-angle tuning, and interface design—positions these materials at the intersection of fundamental quantum physics and practical applications. 2D magnetic vdW heterostructures are poised not only to deepen our understanding of quantum magnetism in reduced dimensions but also to unlock a new era of ultra-low-power, high-efficiency spintronic devices for next-generation memory, logic, and quantum information technologies.

## Figures and Tables

**Table 1:** Comparative summary of intrinsic properties of 2D magnetic materials.

Materials	Electronic Structure	SOC Strength & Orbital Contributions	Magnetic Ordering & Easy Axis	Carrier Mobility (cm^2^/V·s)	Electrical Tunability	Interface Quality & Confinement	Ref.
**CrI_3_**	Insulating E_g_ ~ 1.2 eV	Moderate λ ~ 100–150 meV d orbital (Cr 3d)	FM (T_C_ ~ 61 K bulk, ~45 K monolayer) Easy axis: out-of-plane (c-axis) layer-dependent AFM/FM	Not applicable (insulating ground state)	High: Electrostatic gating controls magnetic order; ionic liquid gating effective	Excellent: Atomically sharp vdW interfaces; strong quantum confinement; air-sensitive	[10,56,71]
**CrBr_3_**	Insulating E_g_ ~ 1.7 eV	Moderate λ ~ 80–120 meV d-orbital (Cr 3d)	FM (T_C_ ~ 34 K monolayer) Easy axis: Out-of-plane interlayer FM coupling	Not applicable (insulating)	High: Gate-tunable magnetism; pressure-sensitive	Excellent: Clean vdW interfaces; 2D Ising behavior; highly air-sensitive	[77,78]
**CGT**	Semiconducting E_g_ ~ 0.7 eV	Moderate-Strong λ ~ 150–200 meV Cr 3d + Te 5p hybridization	FM (T_C_ ~ 61 K bulk) Easy axis: Out-of-plane Robust FM down to monolayer	~10^−2^–10^0^ (hole-doped)	Moderate: Electrostatic doping; strain engineering; magnetic proximity effects	Very Good: Stable vdW interfaces; Moderate air stability; Good for heterostructures	[9,32]
**F_3_GT**	Metallic Itinerant ferromagnet	Moderate λ ~ 100 meV Fe 3d dominant	FM (T_C_ ~ 230 K bulk, ~130 K monolayer) Easy axis: out-of-plane strong perpendicular anisotropy	~10^2^–10^3^ (metallic transport)	Very High: Ionic gating → T_C_ > 300 K; gate-controlled anomalous Hall; proton intercalation	Good: Metallic contacts favorable; some oxidation sensitivity; excellent for spintronics	[24,29]
**F_5_GT**	Metallic Itinerant ferromagnet Higher Fe content	Moderate λ ~ 100–120 meV Fe 3d (enhanced)	FM (T_C_ ~ 310 K bulk, ~270–310 K flake) Easy axis: Out-of-plane Higher T_C_ than F_3_GT More robust FM	~10^2^–10^3^ (metallic, high conductivity)	Very High: Electrostatic gating; enhanced magnetic hardness; stable FM ordering; better than F_3_GT	Very Good: Improved air stability vs F_3_GT; stronger magnetic signals; ideal for RT devices; better device integration	[37,79]
**Graphene**	Semi-metallic Zero-gap semiconductor Dirac fermions	Intrinsically Weak λ ~ 10–50 μeV (pristine) Can be enhanced by proximity C 2p orbitals	Non-magnetic (But: Proximity-induced magnetism possible) Spin transport medium Long spin diffusion length	Ultra-high: ~10^4^–10^6^ (Highest in 2D materials) Ballistic transport	Exceptional: Electrostatic doping (ambipolar); Chemical functionalization; Proximity effects; Twist-angle engineering	Excellent: Perfect vdW interface; atomically thin; transparent electrode; spin transport channel; critical for heterostructures	[80,81,82]
**Mn_3_Si_2_Te_6_**	Semiconductor	Strong λ ~ 150–250 meV Mn 3d + Te 5p hybridization	FM (T_C_ ~ 78 K bulk) Easy axis: In-plane FM due to Mn1 sublattice AFM (Mn1-Mn2 coupling)	Low: ~10^1^–10^2^ Exhibits colossal MR in nanoflakes	Very High: Gate-tunable colossal magnetoresistance; Ultra-low current modulation (~5 A/cm^2^); Sensitive to direct currents; Magnetic proximity effects	Very Good: Clean vdW interfaces; Mechanical exfoliation possible; Platform for chiral orbital moments and spin-torque phenomena	[66,67,83]
**CrSBr**	Semiconducting E_g_ ~ 1.5 eV Excitonic insulator	Weak-Moderate λ ~ 50–80 meV Cr 3d	A-type AFM (T_N_ ~ 132 K) Easy axis: In-plane (a-axis) FM layers, AFM stacking Air-stable!	Not well characterized (semiconducting)	Moderate: Magnetic field control; Optical control of magnetism; Excitonic effects	Excellent: Air-stable. Unique for practical devices; Sharp optical transitions; Good for optoelectronics	[84,85,86]
**CrTe_2_**	Metallic Semi-metallic Itinerant ferromagnet	Moderate-Strong λ ~ 120–180 meV Cr 3d + Te 5p hybridization	FM (T_C_ ~ 310 K bulk, T_C_ ~ 200–300 K thickness-dependent) Easy axis: Out-of-plane Self-intercalation effects	~10^2^–10^3^ (metallic, anisotropic)	Very High: Electrostatic gating; Self-intercalation tuning; Defect engineering; Gate-controlled T_C_	Good: vdW interfaces; Thickness-dependent properties; Air-sensitive; Excellent for RT spintronics	[41,43,50]
**FePS_3_**	Insulating E_g_ ~ 1.5 eV Mott insulator	Weak-Moderate λ ~ 30–60 meV Fe 3d (S/P 3p screening)	AFM (T_N_ ~ 123 K bulk, ~118 K monolayer) Easy axis: In-plane Zigzag-type AFM Strong magnetoelastic coupling	Not applicable (insulating ground state)	Moderate: Magnetic field switching; Optical control (photoexcitation); Pressure-induced transitions; Electrostatic limited	Very Good: Relatively air-stable; Clean vdW interfaces; Strong magneto-optical effects; Good for 2D magnon studies	[87,88]

## Data Availability

No new data were created or analyzed in this study.

## Abbreviations

The following abbreviations are used in this manuscript:

2D	two-dimensional
vdW	van der Waals
CGT	Cr_2_Ge_2_Te_6_
F_3_GT	Fe_3_GaTe_2_
F_5_GT	Fe_5_GeTe_2_
MST	Mn_3_Si_2_Te_6_
3D	three-dimensional
1D	one-dimensional
T_C_	Curie temperature
T_N_	Néel temperature
h-BN	hexagonal boron nitride
SOC	spin–orbit coupling
QAHE	quantum anomalous Hall effect
AHE	anomalous Hall effect
TI	Topological Insulator
Fe	iron
Ge	germanium
Te	tellurium
EB	exchange bias
AFM	antiferromagnetic
FM	ferromagnetic
V_g_	gate voltages
R_xy_	anomalous Hall resistance
ρ_xy_	anomalous Hall resistivity
μ_0_H	magnetic fields
CVD	chemical vapor deposition
MBE	molecular beam epitaxy
CDW	Charge Density Wave
SOT	Spin–Orbit Torque
TMR	Tunneling Magnetoresistance
GMR	Giant Magnetoresistance
DMI	Dzyaloshinskii–Moriya interaction
DOS	Density of States
RHEED	reflection high-energy electron diffraction
MRAM	magnetoresistive random-access memory
UHV	ultra-high vacuum
